# 

**Published:** 1891-08

**Authors:** 


### CONTENTS.

TRANSLATION.

The Oldest Dental Book ... 369
Not so Bad ... 385

COMMUNICATIONS.

Nitrogen a Necessity in the Development of the Teeth ... 386
Dr. W. H. Atkinson ... 388
The Possibilities of Medicine ... 390
The Micro-organisms of Cancer ... 393

SELECTIONS.

Heredity—Some Reflections on it ... 394
Hints for the Profession ... 396
The Use of Rubber Dam ... 397
The Etiology of Tetanus ... 398
The Names of the Grip ... 401
In Simplici Salus ... 403
Cystoma of Jaw ... 405
Items Worth Reading ... 407
Baltimore Letter to Journal of American Medical Association ... 407
Mouth of the Infant at Birth ... 408
Simple Method of Controlling Epistaxis ... 410
Campho-Phenique ... 410
A New Disease ... 411
Brain Rest ... 411
Cardinal Points in Bacteriology ... 412
COMMENCEMENT EXERCISES ... 413

EDITORIAL.

Dental Colleges,—Commencements ... 415
Biographical ... 416

